# Assessment of the Adherence to ESPGHAN 2018 Guidelines in the Neonatal Intensive Care Unit of the Ghent University Hospital: A Retrospective Study

**DOI:** 10.3390/nu15102324

**Published:** 2023-05-16

**Authors:** Joeri De Cloet, Ine Simal, Karel Benoot, Linde Goossens

**Affiliations:** 1 Pharmacy Department, Ghent University Hospital, Corneel Heymanslaan 10, 9000 Ghent, Belgium; joeri.decloet@uzgent.be (J.D.C.); ine.simal@uzgent.be (I.S.); karel.benoot@uzgent.be (K.B.); 2 Neonatology Department, Ghent University Hospital, Corneel Heymanslaan 10, 9000 Ghent, Belgium

**Keywords:** enteral nutrition, parenteral nutrition, guideline recommendations, neonates, practice patterns, premature birth

## Abstract

Parenteral nutrition (PN) is a standard of care for preterm infants in the first postnatal days. The European Society of Paediatric Gastroenterology, Hepatology, and Nutrition (ESPGHAN) has updated their guideline recommendations on PN in 2018. However, data on actual 2018 guideline adherence in clinical practice are sparse. In this retrospective study, conducted at the neonatal intensive care unit (NICU) of Ghent University Hospital, we analyzed the ESPGHAN 2018 PN guideline adherence and growth for 86 neonates admitted to the NICU. Analyses were stratified by birth weight (<1000 g, 1000 to <1500 g, ≥1500 g). We documented the provisions for enteral nutrition (EN) and PN, and we tested the combined EN and PN provisions for ESPGHAN 2018 adherence. The nutrition protocols showed a high adherence to PN guidelines in terms of carbohydrate provisions, yet lipid provisions for EN and PN often exceeded the recommended maximum of 4 g/kg/d; although, PN lipid intakes maxed out at 3.6 g/kg/d. Protein provisions tended to fall below the recommended minimum of 2.5 g/kg/d for preterm infants and 1.5 g/kg/d for term neonates. The energy provisions also tended to fall below the minimum recommendations, especially for neonates with a birth weight (BW) < 1000 g. Over a mean PN duration of 17.1 ± 11.4 d, the median weekly Fenton Z-scores changes for length, weight, and head circumference were positive for all BW groups. Future studies have to assess how protocols adapt to current guidelines, and how this affects short- and long-term growth across different BW groups. In conclusion, the reported findings provide real-world evidence regarding the effect of ESPGHAN 2018 PN guideline adherence, and they demonstrate how standardized neonatal PN solutions can safeguard stable growth during NICU stays.

## 1. Introduction

Worldwide, nearly 1 in 10 births are estimated to be preterm, which is defined as a birth before the gestational age (GA) of 37 weeks [1]. In Europe, 2013 preterm birth rates were estimated to have a prevalence of 6.2% [1]. Additionally, analyses of 19 countries not only revealed a rise in preterm birth rates in most European countries, but also vast international differences; in 2008, preterm birth rates ranged from 5.5% (Finland) to 11.1% (Austria) [2].

The adequate nutrition of preterm infants is indispensable because it establishes physical and neuronal development [3], reduces the risk of adverse outcomes [4,5], and it helps avoiding long-term developmental defects or death [6,7,8]. Conversely, inadequate nutrition can lead to extrauterine growth restriction (EUGR), which refers to the inadequate growth of neonatal intensive care unit (NICU) patients during their first hospital stay [9]. Both short- and long-term prognoses can be worsened by EUGR; for instance, EUGR increases the risk of cardiometabolic alterations during childhood [9]. Due to gut immaturity, the oral or enteral nutrition (EN) of preterm infants is often impossible, insufficient, or contraindicated in preterm infants [10]. As such, parenteral nutrition (PN) is considered to be the standard of care in the first postnatal days of preterm infants [11,12]. In 2018, the European Society of Paediatric Gastroenterology, Hepatology, and Nutrition (ESPGHAN) published new guidelines on pediatric PN, including recommendations for energy [13] and macronutrient provisions [14,15,16]. Nonetheless, several knowledge gaps in pediatric PN demand further data from clinical practice; for instance, the optimal energy or lipid intakes remain unclear [17]. Moreover, in clinical practice, EN intakes are usually gradually introduced until the neonates are ready to be weaned off PN; however, the aforementioned 2018 ESPGHAN guidelines apply solely to PN intakes. Although a recent ESPGHAN position paper provides EN intake recommendations for preterm infants [18], neonatology lacks the clinical data, and thus, the guideline recommendations, concerning the combined use of PN and EN.

In clinical practice, adherence to ESPGHAN PN guidelines is not always guaranteed. A 2013 survey in four European countries assessed adherence to the ESPGHAN 2005 guidelines and found a substantially high prevalence of deviations from guideline recommendations (e.g., 37% of neonatal units provided amino acids on the second, rather than the first, day of life, and 60% provided less than the recommended minimum initial dose to prevent a negative balance) [6]. In 2015, a retrospective analysis of 29 NICUs also detected cumulative deficits in lipid and amino intakes in the first two weeks of life, and it identified the cumulative amino acid intake as an independent variable that is associated with the ∆Z-score for weight at 36 weeks [19]. In addition to acute illnesses and metabolic complications, fluid restrictions are a major obstacle in achieving sufficient PN intakes as they limit PN infusion volumes [20,21]. Especially in this critical population, non-nutritional fluids (e.g., drugs for blood pressure control) are common and can further restrict the PN infusion volumes [22].

Although sufficient PN is crucial for adequate growth in preterm neonates, data concerning the adherence to current nutrition protocols, with regard to ESPGHAN 2018 guidelines, are sparse. Here, we address these knowledge gaps: the objective of this retrospective study was to examine how closely the macronutrient and energy provisions at the NICU at Ghent University Hospital correspond with the respective ESPGHAN 2018 guideline recommendations. As a secondary aim, the weekly growth rates for length, weight, and head circumference were assessed. The analyses were conducted for different body weight (BW) groups to ultimately determine if and how nutrient provisions should be adapted for neonates at the NICU. The results of this study will be used to further adjust the nutrition protocols at Ghent University Hospital, which aims to develop all-in-one PN admixtures, as the use of standardized PN solutions are formulated in accordance with a guideline-recommended approach due to fewer compounding errors and higher levels of patient safety [23].

## 2. Materials and Methods

### 2.1. Study Design, Patient Population, and Data Collection

This retrospective cohort study analyzed 86 neonates that were admitted to the NICU at Ghent University Hospital. Data were collected from 15 December 2013 to 31 December 2014. The sole inclusion criterion was at least 5 consecutive days of PN. Patient data were excluded from analysis if they were transferred to another hospital before they were completely weaned off PN or if PN was interrupted for 14 d or longer. Data were retrieved from medical and nursing records, and lab values were obtained from electronic patient records. Data were collected in anonymized files.

For subgroup analysis, patients were divided into 3 subgroups, in accordance with their BW: <1000 g, 1000 to <1500 g, and ≥1500 g. For the analysis of ESPGHAN 2018 guideline adherence, PN intakes were analyzed for up to 4 weeks. Days were excluded from the analysis if the number of available patients was below 3. Although in clinical practice, EN provisions are usually introduced as early as possible in PN-fed neonates, neither ESPGHAN PN nor EN guidelines provide recommendations on the combined energy and macronutrient intake of PN and EN. As the majority of fluid intake in the first days of life at the NICU are parenteral, we tested the adherence to ESPGHAN PN 2018 guidelines with regard to nutrient provisions, with EN intakes in g/kg/d considered to be equal to PN intakes in g/kg/d for the sake of practicality. All kinds of EN-intakes were considered, including trophic feeding.

### 2.2. Nutrition Protocol

The PN nutrition protocol analyzed in this study was based on the ESPGHAN 2005 recommendations [24], which were last updated in 2013. The protocol utilizes 3 formulations composed of various PN solutions, the use of which has been well-established over the years at Ghent University Hospital:

The first formulation contains glucose (available in different concentrations ranging from 6% to 30%), calcium gluconate (Mini-Plasco 10%^®^, B. Braun, Diegem, Belgium), magnesium sulphate (ampoule 1 g/10 mL Sterop^®^, Brussels, Belgium), water soluble vitamins (Soluvit N^®^, Fresenius Kabi, Schelle, Belgium), and extra vitamin C (Vitamina C ampoule 1 g/5 mL Bayer^®^, Milan, Italy). The second solution provides amino acids (Vaminolact^®^, Fresenius Kabi, Schelle, Belgium), and it is complemented with Tripotassium phosphate (Kaliphos Sterop^®^, Brussels, Belgium), sodium chloride (Mini-Plasco 20%^®^, B. Braun, Diegem, Belgium), and trace elements (Addaven^®^, Fresenius Kabi, Schelle, Belgium). The third formulation provides lipids (SMOFlipid 20%^®^, Fresenius Kabi, Schelle, Belgium) and fat-soluble vitamins (Vitalipid N Infant^®^, Fresenius Kabi, Schelle, Belgium) compounded in a 50 mL opaque syringe.

### 2.3. Variables and Data Management

The primary outcome of this study was adherence to ESPGHAN 2018 guidelines, with regard to macronutrients and energy intake, for each day of PN; this was assessed by examining the fraction of patients whose nutrient/energy provision was in range of the respective minimum and maximum recommended intakes. Secondary outcomes were the weekly changes in Z-scores for weight, height, and head circumference for age.

Analyzed patient demographics were sex, GA, multiple births, and reason for starting/stopping PN, as well as the weight, length, and head circumference at birth and at the start/end of PN. For each patient and day of PN, the volumes of the PN and EN provisions were recorded to calculate the volume fraction (*v*/*v* %) of PN in terms of nutrient provisions. In order to make a comparison with the ESPGHAN 2018 guideline recommendations, the respective intakes from PN and EN were pooled.

Z-scores for weight, length, and head circumference, calculated in accordance with the Fenton growth charts for preterm infants [25], were calculated using the PediTools app [26]. Weekly changes in Z-scores were calculated, as shown in Equation (1):

Weekly change of Z-score = (Z-score at the last day of PN − Z-score at the first day of PN)/time of PN (in weeks)(1)

To test the adherence to ESPGHAN 2018 guidelines, we assessed if the provisions of macronutrients and energy were within the respective ranges, from recommended minimum to maximum. Table 1 summarizes these target ranges. Although guidelines do not state a minimum lipid intake, Ghent University Hospital has a target for minimum lipid intake, which is 1.0 g/kg/d for both preterm and term neonates starting from day 2. Reaching these intake targets, using SMOFlipid 20% as the lipid source, has been well-established at Ghent University Hospital, and the ESPGHAN 2018 recommended minimum supply targets of 0.25 and 0.1 g/kg/d linoleic acid have been reached (preterm and term, respectively) [15].

### 2.4. Bias and Study Size

Common confounders of single-center studies, such as preference of care or center standards, have to be considered for the interpretation of the study. No sample size calculation was performed, and thus, the study size resulted from the number of patient records available, in accordance with the inclusion and exclusion criteria.

### 2.5. Statistical Methods

Parametric variables were summarized as the mean ± standard deviation (SD), or median, along with the first and third quartile. Categorical variables were summarized as counts and relative amounts (%). To check for significant differences between the respective weekly Z-score changes among the 3 BW groups, the equality of variances was first checked using Levene’s test. If the variances were significantly different, the weekly Z-score changes were compared using the Kruskal–Wallis test and pairwise Wilcoxon rank sum tests for post-hoc comparisons. If the variances were not significantly different, the Z-score changes were compared using One-Way ANOVA tests and Tukey’s test for post-hoc comparisons. For post-hoc analyses, *p*-values were adjusted using the Benjamini–Hochberg procedure. The significance level was 0.05.

## 3. Results

### 3.1. Patient Demographics

Table 2 summarizes the patient characteristics for the overall cohort and the different BW groups. Most patients (75, 87.2%) had a GA < 37 weeks, the mean BW was 1659.9 ± 884.3 g, and 55.8% (48) patients were male. The median time from birth to start of PN treatment was 0 d (0–0). The growth parameters at birth, and the median time from birth to PN per BW group, can be found in Table S1.

### 3.2. Reason for PN Initiation

**Figure 1:**
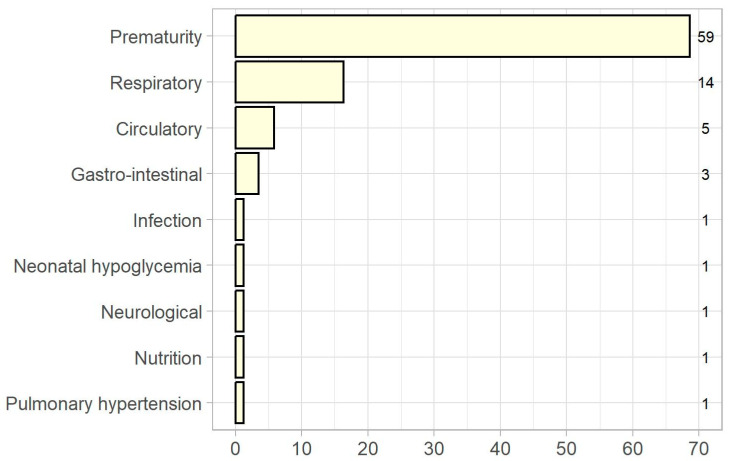
Reason for initiation of parenteral nutrition. Patient numbers are shown on the right.

As Figure 1 shows, prematurity was the main reason for initiation of PN in the cohort (59 patients, 68.6%), followed by respiratory complications (14 patients, 16.3%).

### 3.3. Weekly Changes in Z-Scores

**Figure 2:**
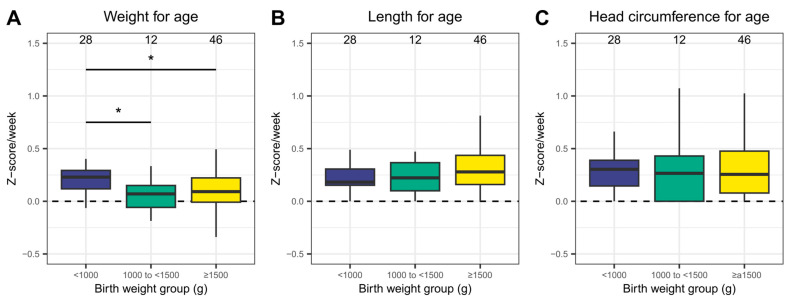
Weekly changes in Z-scores. Z-scores for (**A**) weight and age, (**B**) length and age, and (**C**) head circumference and age were calculated using Fenton growth charts [25]. * *p* < 0.05.

Figure 2 summarizes the weekly changes in Z-scores for weight, length, and head circumference (see Table S2 for a numeric summary). The median weekly changes were positive for all assessments. For weight and age, the weekly Z-score changes were significantly higher for neonates with a BW < 1000 g (0.21 ± 0.12), compared with those with a BW of 1000 to <1500 g (0.07 ± 0.16) or ≥1500 g (0.10 ± 0.31). Moreover, weekly changes for the other Z-scores did not differ between any of the groups.

### 3.4. Carbohydrate Provision

**Figure 3:**
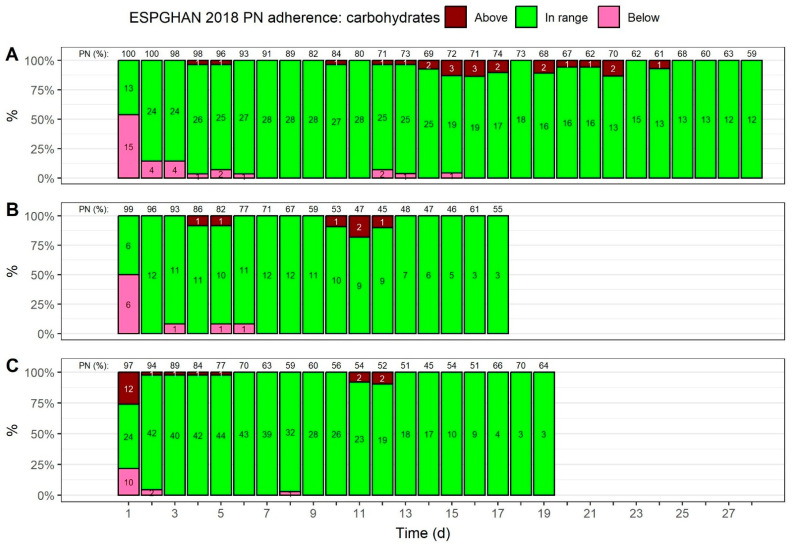
Adherence to ESPGHAN 2018 guideline recommendations, with regard to carbohydrate provisions [14], over time. The total carbohydrate provisions, both for enteral and parenteral nutrition (PN), were used to assess adherence; the mean volume percentage for PN, with regard to glucose provisions, is shown on top. The colors indicate if the provisions fell below the recommended minimum intake, exceeded the maximum intake, or if they were in range. The numbers inside the bars show patient numbers. Adherence was assessed for three birth weight groups: (**A**) <1000 g, (**B**) 1000 to <1500 g, and (**C**) ≥1500 g.

Figure 3 shows how many neonates received a carbohydrate intake that followed ESPGHAN 2018 guideline recommendations over time (see Table S3 for a summary of provided carbohydrates). Regardless of BW, the majority of carbohydrate intakes were in range of the recommendations starting from day 2, and they stayed within range afterwards. On day 1, by way of contrast, 54% of neonates with a BW < 1000 g (Figure 3A), and 50% of neonates with a BW of 1000 to <1500 g (Figure 3B), received a carbohydrate intake that fell below the recommended intake guidelines. For neonates with a BW ≥ 1500 g on day 1, approximately the same number of patients received carbohydrate intakes that exceeded (26%) and fell below (22%) guideline recommendations.

### 3.5. Lipid Provision

**Figure 4:**
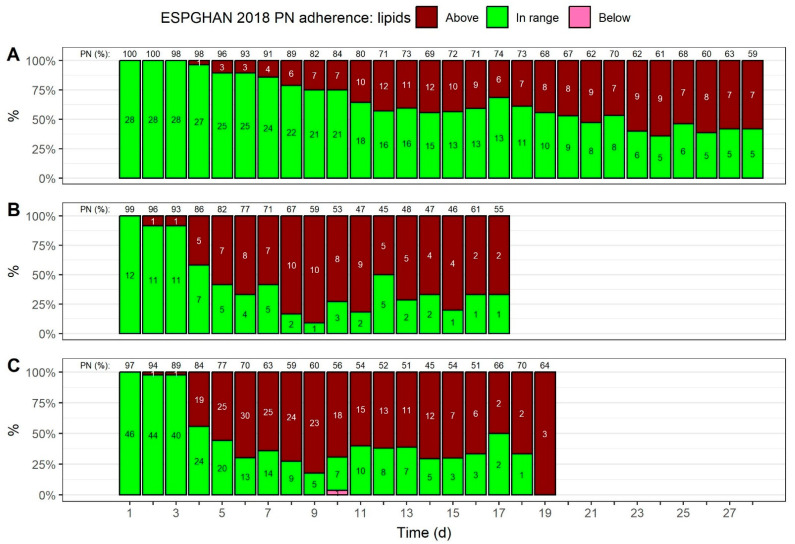
Adherence to ESPGHAN 2018 guideline recommendations, with regard to lipid provisions, [15] over time. The total lipid provisions, for both enteral and parenteral nutrition (PN), were used to assess adherence; the mean volume percentage of PN, with regard to lipid provisions, is shown on top. The colors indicate if the provisions exceeded the recommended maximum intake or if they were in range of 0–4 g/kg/d. Numbers inside the bars show patient numbers. Adherence was assessed for three birth weight groups: (**A**) <1000 g, (**B**) 1000 to <1500 g, and (**C**) ≥1500 g.

Figure 4 shows how many neonates received a lipid intake that followed ESPGHAN 2018 guideline recommendations over time (see Table S4 for a summary of provided lipids). Except for one patient on day 10 in the BW ≥ 1500 g group, all lipid intakes fulfilled the minimum intake goals. However, in all BW groups, the number of patients receiving lipid intakes above the maximum of 4 g/kg/d increased over time, although PN lipid intakes maxed out at 3.6 g/kg/d. The proportion of intakes above the maximum increased gradually in neonates with a BW < 1000 g, from 4% (day 4) to a maximum of 64% (day 24). In contrast, starting from day 5, more than half of the neonates with a BW > 1000 g (Figure 4B,C) received lipid provisions above the maximum until the end of PN.

### 3.6. Amino Acid Provision

**Figure 5:**
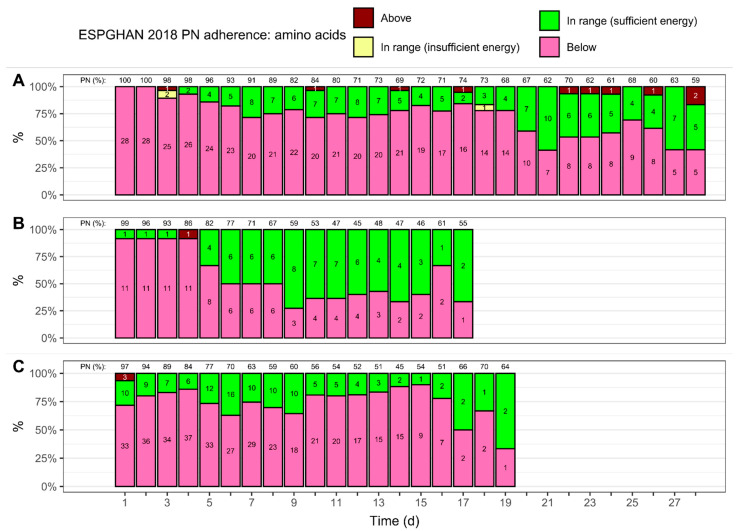
Adherence to ESPGHAN 2018 guideline recommendations, with regard to amino acid provisions [16], over time. The total amino acid provisions, for both enteral and parenteral nutrition (PN), were used to assess adherence; the mean volume percentage of PN, with regard to amino acid provisions, is shown on top. The colors indicate if the provisions fell below the recommended minimum intake, exceeded the maximum, or if they were in range. Insufficient and sufficient energy refers to the intake of non-protein energy. Numbers inside the bars show patient numbers. Adherence was assessed for three birth weight groups: (**A**) <1000 g, (**B**) 1000 to <1500 g, and (**C**) ≥1500 g.

Figure 5 shows how many neonates received an amino acid intake that followed guideline recommendations over time (see Table S5 for a summary of provided amino acids). Overall, most of the intakes fell below the recommended minimum, and intakes that exceeded the recommended maximum were rare. For neonates with a BW < 1000 g (Figure 5A), the amino acid provision fell below the minimum recommendation for most patients over 4 weeks. Of the patients in this group who did receive the recommended amino acid intake, as per ESPGHAN 2018 guidelines, the vast majority of patients also received the recommended amount of non-protein energy. At least half of the patients with a BW of 1000 to <1500 g (Figure 5B) received amino acids in the recommended range, starting from day 6, with the exception of day 16. Of the neonates with a BW ≥ 1500 g (Figure 5C), 63–90% received an amino acid intake that fell below the recommended minimum during the first 16 days of PN.

### 3.7. Energy Provision

**Figure 6:**
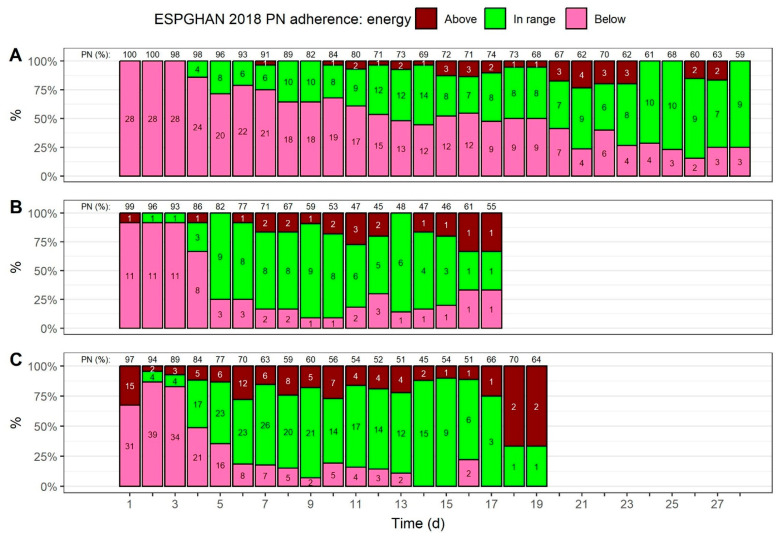
Adherence to ESPGHAN 2018 guideline recommendations, with regard to energy provisions [13], over time. The total energy provisions, for both enteral and parenteral nutrition (PN), were used to assess adherence; the mean volume percentage of PN, with regard to energy provisions, is shown on top. The colors indicate if the provision fell below the recommended minimum intake, exceeded the maximum, or if they were in range. Numbers inside the bars show patient numbers. Adherence was assessed for three birth weight groups: (**A**) <1000 g, (**B**) 1000 to <1500 g, and (**C**) ≥1500 g.

Figure 6 shows how many neonates received energy from all macronutrient intakes over time, in accordance with guideline recommendations (see Table S6 for a summary of provided energy). All three BW groups have two distinctive features in common: first, energy intakes mostly fell below the recommended minimum for the first three days. Second, the proportion of neonates with energy intakes that adhered to the guidelines increased after this point. This increase was more pronounced for neonates with a BW > 1000 g (Figure 6B,C), as compared with neonates that had a lower BW (Figure 6A). In the period from day 5 to day 15, the proportion of energy intakes that adhered to the guidelines ranged from 50–86% for neonates with a BW of 1000 to <1500 g (Figure 6B). For neonates with a BW ≥ 1500 g, 51–90% of patients received energy intakes that adhered to the guidelines in the period from day 5 to day 15.

### 3.8. Volume Percentage of PN Regarding Nutrient Provisions over Time

As shown in Figure 3, Figure 4, Figure 5 and Figure 6, above the columns, regardless of the BW, the mean volume percentage of PN, regarding nutrient provisions, started close to 100%, and declined gradually (see Table S7 for a summary). The most apparent difference between the BW groups is that the relative amount of PN declined more slowly in neonates with a BW < 1000 g; after 2 weeks, the amount declined to 69%, whereas less than 50% of the nutrient intake was parenteral in neonates with higher BWs. In neonates with a BW < 1000 g, the PN proportion in terms of nutrient provisions was at least 59% across the four weeks of observation.

## 4. Discussion

By analyzing the proportion of patients whose macronutrient and energy intakes followed ESPGHAN 2018 recommendations, we found that guideline adherence, with regard to macronutrient and energy provisions, showed certain patterns across all three BW groups: (1) carbohydrate provisions were in range for the vast majority of patients starting from day 2, although half of the patients with a BW < 1500 g received a carbohydrate intake that fell below minimum recommendations on the first day of PN; (2) lipid provisions tended to exceed the maximum recommendations over time, and this was more pronounced in neonates with a BW ≥ 1000 g; (3) amino acid provisions generally fell below recommended levels, especially in neonates with a BW < 1000 g; and (4) energy provisions fell below the minimum recommendations for the first 3 days of PN, although, the number of patients receiving energy provisions recommended by the guidelines increased slowly, with a stronger increase in neonates with a BW ≥ 1000 g.

For an appropriate interpretation of these results, certain strengths and limitations of this study should be noted. The single-center nature of this study is a double-edged sword; on the one hand, the results are not confounded by different clinical practices across centers, on the other hand, we cannot dismiss the center bias. Moreover, sample sizes are also limited, especially for neonates with a BW of 1000 to <1500 g. The small sample sizes also limit the possibility of further stratification with regard to longitudinal analyses (e.g., by pathology: during the patients’ stay, congenital heart disease occurred only in 3 patients (3.5%), and catheter-related bloodstream infections occurred in 8 patients (9.3%)). Additionally, although respiratory support was more common, occurring in 32 patient cases (37.2%), only three of those involved patients with a BW of 1000 to <1500 g (<1000 g: 16, ≥1500 g: 13). In terms of generalizability, the results mainly apply to neonates whose primary PN indication is prematurity (68.8% of patients in this study). We acknowledge that combining PN and EN intakes to analyze ESPGHAN 2018 PN guidelines is a compromise; on the one hand, current guideline recommendations lack clear recommended intakes for the combined use of EN and PN in preterm neonates, but on the other hand, EN feeds are introduced as soon as possible in clinical practice, so the time that a patient is on total PN (TPN) is often extremely short. In a real-world setting, compromises for assessing PN guideline adherence are practically unavoidable for the majority of patients. For instance, a cohort study by Boscarino et al. has defined TPN as a total PN energy intake of more than 70% during the first week of life [27]. To account for EN intakes, and to allow readers to draw meaningful conclusions from these results, this study provides a PN fraction for each given day. Given that there is a severe lack of data on how the combined use of PN and EN relates to clinical outcomes, this is a major strength of this study. As an alternative approach to the equalization of PN and EN volume provisions, future adaptions of this approach may consider adjustments to reflect the less efficient metabolization of EN compared with PN. For instance, the ESPGHAN 2018 guidelines state that the gross energy content of 1 g of amino acids (PN) is about 10% lower than that of 1 g of protein (EN) [13]; therefore, to calculate the energy provisions, with regard to amino acids, volume equivalent EN intakes could be adjusted to 110% of the energy provisions of the PN amino acid intakes. Moreover, regarding the aforementioned cohort study by Boscarino et al. [27], one may also consider setting a certain limit on the PN intake that is required to qualify for inclusion in the dataset.

Despite the differences between BW groups in terms of ESPGHAN 2018 guideline adherence, we observed positive Z-score developments across all BW groups, thus indicating that the nutrition protocols were sufficient to achieve growth. The positive growth rates contrast with the negative growth rates in the first weeks of life from other observational studies on preterm development [28,29,30]. Differences between BW among various cohorts might explain these differences. For instance, although Izquierdo Renau et al. [28] report negative weight developments 28 d after birth for preterm infants, the mean BW of 1200 ± 360 g in that cohort was notably lower than in our cohort (1659.9 ± 884.3 g). However, it is estimated that BW accounts for only 7% of growth variation in preterm neonates, whereas nutrition accounts for about 45% [31]. A cohort study by Westin et al. clearly demonstrates the effect of different nutrition protocols on growth, as nutrition protocols have been continuously updated between 2004 and 2011. These include protocols that concern energy and macronutrient intakes in preterm infants, and improvements have been made, particularly when compared with the period 2004–2005; indeed, neonates in later periods received an earlier and higher provision of lipids, proteins, and energy in the first 28 d of life, thus leading to significantly higher gains in length, weight, and head circumference as per the 0.3–0.5 standard deviation scores. Additionally, linear mixed-effects model analyses by Asbury et al. [32] have shown that early and sufficient intakes of macronutrients have positive effects on Z-scores, independent of demographics, acuity, and morbidity. Thus, we cannot exclude the possibility that a greater adherence to ESPGHAN 2018 guidelines, regarding amino acid and energy provisions, would result in higher weekly Z-score developments, or significant differences between BW groups.

In terms of adherence to ESPGHAN 2018 carbohydrate recommendations [13], the analyses indicate sufficient carbohydrate provisions from day 2 for all BW groups. Provisions in range of the recommendations should reduce the risk of overfeeding and hyperglycemia [14]. In fact, the latter occurred in only eight patients (9.3%) for a duration of three or more consecutive days.

Regarding lipid intakes, the current protocols may be adapted to avoid the increasing number of neonates with intakes exceeding the recommended maximum of 4 g/kg/d [15], starting from day 4, especially for neonates with a BW > 1000 g. However, PN provisions maxed out at 3.6 g/kg/d. In a clinical practice at Ghent University Hospital, triglyceride levels are measured on a weekly basis and lipid provisions are reduced if hypertriglyceridemia is detected. In this cohort, only one patient had hypertriglyceridemia that persisted for three consecutive days, despite several patients having provisions above the recommended maximum. A multiple logistic regression model by Giretti et al. [33] might explain this apparent lack of association; although each additional intravenous lipid intake of 1 g/kg/d increased the risk of hypertriglyceridemia by 96%, for each week of GA, and for each g/kg/d of amino acid intake, the risk of hypertriglyceridemia was reduced by 12% and 19%, respectively [33]. Moreover, the use of fish oil emulsions was associated with a significantly lower risk of hypertriglyceridemia (a reduction of 38%), which the authors traced back to lipogenesis downregulation by fish oil emulsions. Ultimately, although our nutrition protocols may have to be adapted to not exceed ESPGHAN 2018 guideline recommendations, we can corroborate the findings of other cohort studies on the safe use of fish oil lipid emulsions in terms of hypertriglyceridemia [33,34].

The analyses of amino acid administration showed a high prevalence of provisions that fell below the minimum ESPGHAN 2018 recommendations across all BW groups, especially in the BW < 1000 g and BW ≥ 1500 g groups. If the amino acid provisions can be increased, it may benefit growth rates. Linear mixed-effects model analyses showed that early and high protein intakes in the first week of life ameliorate weight decline after birth, and from day 9 onward, they are significantly associated with greater increases in weight, length, and head circumference [32]. Although the optimal glucose and lipid intakes to safeguard protein accretion and growth are unknown [16], the nutrition protocol analyzed in this study showed that insufficient non-protein intakes were a rare exception (three cases on two days). However, if amino acid provision guidelines recommend an increase in the next protocol update, this finding has to be reassessed.

Furthermore, we would also have to reassess how the energy intakes comply with current guideline recommendations if lipid provisions are reduced and amino acid provisions are increased. This is especially important in neonates with a BW < 1000 g, who seem to be especially susceptible to energy provisions that fall below the minimum recommended amount. Nonetheless, although insufficient energy intakes bear the risk of impaired growth [13], all weekly median Z-score changes were positive. Given the lack of data after patients left the NICU, it remains unclear if long-term developments differed between the BW groups. The occurrence of EUGR in this cohort might provide an estimate of how many patients had a higher risk of developing growth defects in early childhood. Although the definition of EUGR is debated [11], an analysis of over 800 very low birth weight or preterm neonates in an Italian cohort found that, compared with a cross-sectional definition, a longitudinal definition of EUGR was a better predictor for poorer growth outcomes 24–30 months after discharge. Furthermore, Peila et al. 2020 defined EUGR as a weight loss > 1 Z-score (Italian Neonatal Study charts) between birth and a given time (36 and 40 weeks of GA, discharge, 28 days) [35]. However, applying this definition to the ∆Fenton Z-scores from day 1 to discharge in our cohort did not provide any indication for long-term growth deficits, as none of the patients had EUGR. A possible explanation for this observation might be that only longer durations of (T)PN are associated with EUGR occurrence. For instance, a prospective study by Wang et al. showed that the median PN duration was significantly higher for EUGR patients at 24 d compared with non-EUGR patients at 17 d [36], with the latter closely resembling the mean PN duration in our study. Additionally, the authors found longer PN durations to be an independent risk factor for EUGR occurrence [36], thus echoing the findings of similar EUGR studies [37,38,39]. If changes in future nutrition protocols at UZ Ghent increase the (T)PN duration, the potential effect on EUGR should be assessed, including follow-ups after NICU discharge.

We observed that patients in the lower BW groups showed a slower reduction in terms of the PN fraction, regarding the nutrition provision, which is indicative of the less stable condition of these patients. An observational study by Immeli et al. showed that for very low birth weight infants, the length of the transition phase is negatively correlated with the cumulative energy and macronutrient intakes at 28 d, and longer transition phases are associated with slower postnatal growth rates [40]. This may be one more reason to increase protein and energy provisions in these patients.

The results of this study may inform further adjustments to the nutrition protocols of the NICU at Ghent University Hospital, which mainly recommend increases to amino acid provisions and lower lipid provisions. Due to their well-established usage and good effect on growth, the nutrition regimen will continue to utilize standardized PN solutions. The next step will be to assess the possibilities of introducing all-in-one PN admixtures in order to reduce compounding errors and increase patient safety.

## 5. Conclusions

In this study, we report the results of the assessment of the current nutrition protocols at Ghent University Hospital, as per ESPGHAN 2018 PN guidelines. We found that future protocol adaptations should lower lipid provisions and increase both protein and energy provisions. In addition, our findings provide relevant insights into neonatal PN practices at a Belgian NICU on a more general level. The reported approach of considering both PN and EN intakes does not only provide real-world data on an under-researched topic of neonatal nutrition, it might also serve as a template for other centers who want to assess their PN guideline adherence pragmatically. Importantly, even with deviations from current PN guidelines, positive Z-score developments over a mean PN duration of 17.1 ± 11.4 days were possible across different BW groups. In that regard, this study also provides real-world evidence on how the use of standardized PN solutions can safeguard the development of critically ill neonates during their NICU stay. Future studies should check how adaptations of the protocol will reflect short- and long-term growth, guideline adherence, and safety outcomes.

## Figures and Tables

**Table 1:** Overview of recommended minimum and maximum intakes defining the target ranges for macronutrient and energy intakes. Recommended values were taken from the respective guidelines issued by the European Society for Paediatric Gastroenterology, Hepatology, and Nutrition [13,14,15,16]. The minimum lipid targets correspond with the recommended minimum intake of linoleic acid [15].

Gestational Age	Nutrient	Minimum Day 1	Maximum Day 1	Minimum Day 2+	Maximum Day 2+
<37 weeks	Carbohydrates (g/kg/d)	5.8	11.5	5.8	17.3
	Lipids (g/kg/d)	-	-	1.0	4
	Amino acids (g/kg/d)	1.5	2.5	2.5	3.5
	Non-protein energy (kcal/kg/d)	-	-	>65	>65
	Energy (kcal/kg/d)	45.0	55.0	90.0	120
≥37 weeks	Carbohydrates (g/kg/d)	3.6	7.2	3.6	17.3
	Lipids (g/kg/d)	-	-	1.0	4
	Amino acids (g/kg/d)	1.5	3.0	1.5	3.0
	Energy (kcal/kg/d)	45.0	50.0	75	85

**Table 2:** Summary of patient characteristics for the overall cohort, stratified by birth weight (BW). SD standard deviation, PN parenteral nutrition.

Parameter	Overall	BW < 1000 g	BW of 1000 to <1500 g	BW ≥ 1500 g
Patients, n (%)	86 (100)	28 (32.6)	12 (16.3)	46 (51.2)
Sex male, n (%)	48 (55.8)	18 (64.3)	4 (33.3)	26 (56.5)
Gestational age < 37 weeks, n (%)	75 (87.2)	28 (100)	12 (100)	35 (76.1)
Multiple birth yes, n (%)	32 (37.2)	9 (32.1)	6 (50)	17 (37.0)
Duration of PN, d, mean ± SD	17.1 ± 11.4	27.4 ± 13.8	14.9 ± 5.6	11.4 ± 4.7
Weight at start of PN, g, mean ± SD	1655 ± 873.4	792.8 ± 107.4	1287.7 ± 161.2	2275.7 ± 731.5
Weight at end of PN, g, mean ± SD	1769.5 ± 872.3	973.4 ± 163.5	1345.7 ± 183.2	2364.6 ± 780.2
Length at start of PN, cm, mean ± SD	40.6 ± 6.1	33.8 ± 2	38.6 ± 1.2	45.3 ± 3.8
Length at end of PN, cm, mean ± SD	42.1 ± 5.9	35.7 ± 2.1	40 ± 1.3	46.6 ± 3.9
Head circumference at start of PN, cm, mean ± SD	28.1 ± 4.1	23.6 ± 1.6	27 ± 1.1	31.1 ± 2.7
Head circumference at end of PN, cm, mean ± SD	29.1 ± 3.8	25.2 ± 1.6	27.7 ± 1.6	31.9 ± 2.6

## Data Availability

Due to patient confidentiality, we are unable to publish the raw data for this study.

## Supplementary Materials

The following supporting information can be downloaded at: https://www.mdpi.com/article/10.3390/nu15102324/s1, Table S1: Patient growth parameters at birth and median time from birth to parenteral nutrition (PN), stratified by birth weight (BW), Table S2: Summary of weekly changes of Z-scores for age, Table S3: Carbohydrate provision in neonates stratified by birth weight (BW), Table S4: Lipid provision in neonates stratified by birth weight (BW), Table S5: Amino acid provision in neonates stratified by birth weight (BW), Table S6: Energy provision in neonates stratified by birth weight (BW), Table S7: Parenteral nutrition fraction (*v*/*v* %) in the nutrient provision, stratified by birth weight (BW).
