# Supplementary material for: Assessment of the Adherence to ESPGHAN 2018 Guidelines in the Neonatal Intensive Care Unit of the Ghent University Hospital: A Retrospective Study
Source: Nutrients. 2023 May 16;15(10):2324. doi: 10.3390/nu15102324 (PMC10221736; doi:10.3390/nu15102324)
Supplement: Supplementary file 1 [file nutrients-15-02324-s001.zip › Table_S1.pdf]

Table S1. Patient growth parameters at birth and median time from birth to parenteral nutrition (PN), stratified by birth weight (BW). SD standard deviation, IQR interquartile range.

Parameter	Overall	BW < 1000 g	BW of 1000 to < 1500 g	BW ≥ 1500 g
Birth weight, g, mean ± SD	1659.9 ± 884.3	792.8 ± 107.4	1287.7 ± 161.2	2284.8 ± 748.2
Birth length, cm, mean ± SD	40.6 ± 6	33.8 ± 2	38.6 ± 1.2	45.2 ± 3.7
Birth head circumference, cm, mean ± SD	28.1 ± 4	23.6 ± 1.6	27 ± 1.1	31.1 ± 2.6
Time from birth to PN, days, median (IQR)	0.0 (0.0–0.0)	0 (0.0–0.0)	0.0 (0.0–0.0)	0.0 (0.0–1.8)
