# Supplementary material for: Assessment of the Adherence to ESPGHAN 2018 Guidelines in the Neonatal Intensive Care Unit of the Ghent University Hospital: A Retrospective Study
Source: Nutrients. 2023 May 16;15(10):2324. doi: 10.3390/nu15102324 (PMC10221736; doi:10.3390/nu15102324)
Supplement: Supplementary file 1 [file nutrients-15-02324-s001.zip › Table_S2.pdf]

Table S2. Summary of weekly changes of Z-scores for age. Calculation based on Fenton growth charts [1]. Min minimum, Q25 first quartile, Q75 third quartile, Max maximum, SD standard deviation.

Weekly Fenton Z-score change	Birth weight (g)	Min	Q25	Median	Q75	Max	Mean	SD
Weight	<1000	-0.06	0.12	0.23	0.29	0.40	0.21	0.12
Length	<1000	0.00	0.15	0.18	0.31	0.49	0.22	0.13
Head circumference	<1000	0.00	0.15	0.30	0.39	0.66	0.29	0.18
Weight	1000 to <1500	-0.19	-0.06	0.07	0.15	0.33	0.07	0.16
Length	1000 to <1500	0.00	0.10	0.22	0.37	0.47	0.23	0.16
Head circumference	1000 to <1500	-1.14	0.00	0.27	0.43	1.07	0.21	0.52
Weight	≥1500	-0.53	-0.01	0.09	0.22	1.32	0.10	0.31
Length	≥1500	0.00	0.16	0.28	0.44	1.21	0.33	0.27
Head circumference	≥1500	0.00	0.08	0.26	0.48	1.18	0.33	0.31

References

1 Fenton TR, Kim JH. A systematic review and meta-analysis to revise the Fenton growth chart for preterm infants. *BMC Pediatr* 2013;13:59. doi:10.1186/1471-2431-13-59.
