# Supplementary material for: Assessment of the Adherence to ESPGHAN 2018 Guidelines in the Neonatal Intensive Care Unit of the Ghent University Hospital: A Retrospective Study
Source: Nutrients. 2023 May 16;15(10):2324. doi: 10.3390/nu15102324 (PMC10221736; doi:10.3390/nu15102324)
Supplement: Supplementary file 1 [file nutrients-15-02324-s001.zip › Table_S3.pdf]

Table S3. Carbohydrate provision in neonates stratified by birth weight (BW). Intake data (in g/kg/d) show mean and standard deviation.

	**BW < 1000 g**		**BW of 1000 to < 1500 g**		**BW ≥ 1500 g**	
**Day**	**N**	**g/kg/d**	**N**	**g/kg/d**	**N**	**g/kg/d**
1	28	5.2 ± 2.6	12	4.8 ± 3.2	46	8.1 ± 3.6
2	28	8.1 ± 2.0	12	8.2 ± 2.2	45	9.0 ± 2.4
3	28	8.7 ± 2.8	12	8.6 ± 2.4	41	10.3 ± 2.3
4	28	9.7 ± 3.2	12	10.5 ± 3.3	43	11.1 ± 2.3
5	28	11.0 ± 2.9	12	11.8 ± 3.0	45	11.8 ± 2.4
6	28	11.2 ± 2.7	12	11.6 ± 3.0	43	12.5 ± 2.3
7	28	11.6 ± 2.7	12	12.1 ± 2.9	39	11.9 ± 2.4
8	28	11.9 ± 2.4	12	11.8 ± 2.7	33	12.1 ± 2.8
9	28	11.3 ± 2.2	11	11.7 ± 2.7	28	11.9 ± 1.8
10	28	11.8 ± 2.8	11	13.3 ± 3.5	26	12.5 ± 2.1
11	28	12.1 ± 2.5	11	13.2 ± 2.5	25	12.7 ± 2.6
12	28	11.3 ± 3.7	10	13.7 ± 3.9	21	12.9 ± 3.2
13	27	12.3 ± 3.1	7	12.9 ± 3.1	18	12.8 ± 2.3
14	27	12.8 ± 3.9	6	13.0 ± 3.6	17	13.0 ± 1.7
15	23	12.4 ± 3.2	5	12.3 ± 3.5	10	12.7 ± 2.1
16	22	12.8 ± 3.2	3	12.2 ± 4.5	9	13.1 ± 1.2
17	19	12.8 ± 3.3	-	-	4	13.0 ± 1.4
18	18	12.1 ± 2.8	-	-	3	13.1 ± 1.8
19	18	12.1 ± 3.1	-	-	3	11.5 ± 1.8
20	17	13.0 ± 3.2	-	-	-	-
21	17	13.9 ± 2.4	-	-	-	-
22	15	14.3 ± 4.2	-	-	-	-
23	15	13.8 ± 2.1	-	-	-	-
24	14	13.1 ± 1.9	-	-	-	-
25	13	13.1 ± 2.6	-	-	-	-
26	13	13.1 ± 2.1	-	-	-	-
27	12	13.9 ± 1.4	-	-	-	-
28	12	13.5 ± 1.4	-	-	-	-
