# Supplementary material for: Assessment of the Adherence to ESPGHAN 2018 Guidelines in the Neonatal Intensive Care Unit of the Ghent University Hospital: A Retrospective Study
Source: Nutrients. 2023 May 16;15(10):2324. doi: 10.3390/nu15102324 (PMC10221736; doi:10.3390/nu15102324)
Supplement: Supplementary file 1 [file nutrients-15-02324-s001.zip › Table_S4.pdf]

Table S4. Lipid provision in neonates stratified by birth weight (BW). Intake data (in g/kg/d) show mean and standard deviation.

	BW < 1000 g		BW of 1000 to < 1500 g		BW ≥ 1500 g	
**Day**	**N**	**g/kg/d**	**N**	**g/kg/d**	**N**	**g/kg/d**
1	28	0.0 ± 0.0	12	0.3 ± 0.7	46	0.7 ± 1.0
2	28	0.7 ± 0.5	12	1.1 ± 1.3	45	1.8 ± 0.9
3	28	1.5 ± 0.7	12	2.2 ± 1.3	41	2.8 ± 0.7
4	28	2.3 ± 1.0	12	3.7 ± 1.8	43	3.7 ± 1.0
5	28	2.9 ± 1.1	12	4.4 ± 1.1	45	4.1 ± 1.4
6	28	2.6 ± 1.1	12	4.6 ± 1.2	43	4.7 ± 1.3
7	28	2.5 ± 1.5	12	4.8 ± 1.2	39	4.5 ± 1.3
8	28	2.7 ± 1.6	12	5.2 ± 1.3	33	4.8 ± 1.6
9	28	3.1 ± 1.5	11	5.6 ± 1.5	28	5.2 ± 1.4
10	28	2.9 ± 1.9	11	4.5 ± 0.8	26	4.7 ± 1.8
11	28	4.7 ± 6.6	11	4.8 ± 0.9	25	4.3 ± 1.5
12	28	3.5 ± 1.7	10	4.2 ± 0.9	21	4.5 ± 1.4
13	27	3.4 ± 1.4	7	4.3 ± 1.0	18	4.7 ± 1.1
14	27	3.5 ± 1.5	6	4.3 ± 1.0	17	4.7 ± 1.0
15	23	3.4 ± 1.6	5	4.5 ± 1.3	10	4.5 ± 0.9
16	22	3.6 ± 1.5	3	4.6 ± 1.8	9	4.4 ± 0.9
17	19	3.6 ± 1.6	3	4.9 ± 2.1	4	4.1 ± 0.8
18	18	3.7 ± 1.5	-	-	3	4.8 ± 0.7
19	18	4.0 ± 1.5	-	-	3	5.0 ± 0.8
20	17	4.0 ± 1.6	-	-	-	-
21	17	4.2 ± 1.4	-	-	-	-
22	15	3.8 ± 1.5	-	-	-	-
23	15	4.2 ± 1.5	-	-	-	-
24	14	3.9 ± 1.5	-	-	-	-
25	13	3.8 ± 1.7	-	-	-	-
26	13	4.2 ± 1.5	-	-	-	-
27	12	3.9 ± 1.7	-	-	-	-
28	12	4.0 ± 1.6	-	-	-	-
