# Supplementary material for: Assessment of the Adherence to ESPGHAN 2018 Guidelines in the Neonatal Intensive Care Unit of the Ghent University Hospital: A Retrospective Study
Source: Nutrients. 2023 May 16;15(10):2324. doi: 10.3390/nu15102324 (PMC10221736; doi:10.3390/nu15102324)
Supplement: Supplementary file 1 [file nutrients-15-02324-s001.zip › Table_S5.pdf]

Table S5. Amino acid provision in neonates stratified by birth weight (BW). Intake data (in g/kg/d) show mean and standard deviation.

	**BW < 1000 g**		**BW of 1000 to < 1500 g**		**BW ≥ 1500 g**	
**Day**	**N**	**g/kg/d**	**N**	**g/kg/d**	**N**	**g/kg/d**
1	28	0.5 ± 0.3	12	0.6 ± 0.5	46	1.2 ± 0.9
2	28	1.4 ± 0.4	12	1.3 ± 0.7	45	1.5 ± 0.4
3	28	1.8 ± 0.7	12	1.6 ± 0.4	41	1.8 ± 0.4
4	28	1.9 ± 0.4	12	2.1 ± 1.0	43	1.9 ± 0.4
5	28	2.0 ± 0.5	12	2.3 ± 0.6	45	2.0 ± 0.5
6	28	2.0 ± 0.5	12	2.3 ± 0.7	43	2.1 ± 0.5
7	28	2.1 ± 0.5	12	2.3 ± 0.7	39	1.9 ± 0.6
8	28	2.1 ± 0.5	12	2.4 ± 0.7	33	2.0 ± 0.6
9	28	2.1 ± 0.5	11	2.4 ± 0.7	28	2.0 ± 0.6
10	28	2.1 ± 0.6	11	2.5 ± 0.6	26	1.9 ± 0.5
11	28	2.1 ± 0.6	11	2.6 ± 0.5	25	1.9 ± 0.6
12	28	2.1 ± 0.6	10	2.6 ± 0.6	21	2.0 ± 0.5
13	27	2.2 ± 0.6	7	2.5 ± 0.6	18	1.9 ± 0.5
14	27	2.1 ± 0.6	6	2.5 ± 0.8	17	1.8 ± 0.5
15	23	2.2 ± 0.5	5	2.5 ± 0.9	10	1.8 ± 0.5
16	22	2.1 ± 0.6	3	2.1 ± 0.7	9	2.0 ± 0.6
17	19	2.2 ± 0.6	3	2.1 ± 0.9	4	2.1 ± 0.6
18	18	2.1 ± 0.5	-	-	3	2.1 ± 0.5
19	18	2.1 ± 0.5	-	-	3	2.1 ± 0.3
20	17	2.2 ± 0.6	-	-	-	-
21	17	2.6 ± 0.6	-	-	-	-
22	15	2.5 ± 0.8	-	-	-	-
23	15	2.5 ± 0.7	-	-	-	-
24	14	2.5 ± 0.6	-	-	-	-
25	13	2.3 ± 0.6	-	-	-	-
26	13	2.5 ± 0.6	-	-	-	-
27	12	2.5 ± 0.5	-	-	-	-
28	12	2.7 ± 0.7	-	-	-	-
