# Supplementary material for: Assessment of the Adherence to ESPGHAN 2018 Guidelines in the Neonatal Intensive Care Unit of the Ghent University Hospital: A Retrospective Study
Source: Nutrients. 2023 May 16;15(10):2324. doi: 10.3390/nu15102324 (PMC10221736; doi:10.3390/nu15102324)
Supplement: Supplementary file 1 [file nutrients-15-02324-s001.zip › Table_S6.pdf]

Table S6. Energy provision in neonates stratified by birth weight (BW). Intake data (in kcal/kg/d) show mean and standard deviation.

	BW < 1000 g		BW of 1000 to < 1500 g		BW ≥ 1500 g	
**Day**	**N**	**kcal/kg/d**	**N**	**kcal/kg/d**	**N**	**kcal/kg/d**
1	28	22.8 ± 11.3	12	23.7 ± 19.4	46	43.6 ± 23.4
2	28	43.7 ± 10.2	12	47.9 ± 22.3	45	58.1 ± 15.2
3	28	55.2 ± 16.2	12	61.0 ± 22.0	41	73.6 ± 10.0
4	28	68.7 ± 16.5	12	83.8 ± 32.4	43	85.5 ± 13.1
5	28	78.7 ± 17.0	12	95.6 ± 19.3	45	91.5 ± 17.8
6	28	76.3 ± 16.1	12	97.3 ± 19.8	43	100.6 ± 16.1
7	28	77.5 ± 21.1	12	100.5 ± 20.3	39	95.4 ± 18.9
8	28	80.0 ± 19.2	12	103.4 ± 21.1	33	99.5 ± 19.5
9	28	81.3 ± 18.9	11	107.0 ± 20.9	28	102.5 ± 14.5
10	28	82.2 ± 26.6	11	103.6 ± 19.2	26	99.9 ± 16.6
11	28	85.6 ± 24.1	11	106.6 ± 13.6	25	97.2 ± 16.5
12	28	85.2 ± 23.9	10	103.3 ± 23.3	21	100 ± 18.8
13	27	88.8 ± 20.2	7	100.2 ± 20.5	18	101 ± 12.9
14	27	91.2 ± 20.4	6	100.2 ± 23.5	17	101.7 ± 7.8
15	23	89.1 ± 23.3	5	99.6 ± 25.3	10	98.2 ± 9.5
16	22	92.3 ± 22.9	3	98.6 ± 34.5	9	99.8 ± 11.7
17	19	92.2 ± 23.7	3	99.6 ± 37.6	4	97.3 ± 10.8
18	18	90.5 ± 18.4	-	-	3	103.7 ± 9.8
19	18	92.7 ± 19.8	-	-	3	99.7 ± 12.7
20	17	96.5 ± 22.0	-	-	-	-
21	17	103.6 ± 17.7	-	-	-	-
22	15	101.8 ± 27.7	-	-	-	-
23	15	102.6 ± 18.4	-	-	-	-
24	14	97.8 ± 14.8	-	-	-	-
25	13	96.2 ± 18.7	-	-	-	-
26	13	100.0 ± 16.3	-	-	-	-
27	12	101.0 ± 16.1	-	-	-	-
28	12	100.6 ± 14.8	-	-	-	-
