# Supplementary material for: Assessment of the Adherence to ESPGHAN 2018 Guidelines in the Neonatal Intensive Care Unit of the Ghent University Hospital: A Retrospective Study
Source: Nutrients. 2023 May 16;15(10):2324. doi: 10.3390/nu15102324 (PMC10221736; doi:10.3390/nu15102324)
Supplement: Supplementary file 1 [file nutrients-15-02324-s001.zip › Table_S7.pdf]

Table S7. Parenteral nutrition fraction (v/v%) in the nutrient provision, stratified by birth weight (BW). Data show mean and standard deviation (SD).

	BW < 1000 g		BW of 1000 to < 1500 g		BW ≥ 1500 g	
**Day**	**N**	**%**	**N**	**%**	**N**	**%**
1	28	100 ± 0	12	99 ± 2	46	97 ± 11
2	28	100 ± 1	12	96 ± 7	45	94 ± 12
3	28	98 ± 3	12	93 ± 9	41	89 ± 13
4	28	98 ± 4	12	86 ± 16	43	84 ± 16
5	28	96 ± 6	12	82 ± 13	45	77 ± 19
6	28	93 ± 9	12	77 ± 15	43	70 ± 22
7	28	91 ± 12	12	71 ± 17	39	63 ± 23
8	28	89 ± 14	12	67 ± 19	33	59 ± 23
9	28	82 ± 23	11	59 ± 24	28	60 ± 23
10	28	84 ± 19	11	53 ± 26	26	56 ± 24
11	28	80 ± 23	11	47 ± 27	25	54 ± 26
12	28	71 ± 31	10	45 ± 28	21	52 ± 28
13	27	73 ± 27	7	48 ± 30	18	51 ± 27
14	27	69 ± 31	6	47 ± 31	17	45 ± 27
15	23	72 ± 30	5	46 ± 34	10	54 ± 26
16	22	71 ± 30	3	61 ± 37	9	51 ± 30
17	19	74 ± 27	3	55 ± 43	4	66 ± 27
18	18	73 ± 25	-	-	3	70 ± 13
19	18	68 ± 29	-	-	3	64 ± 15
20	17	67 ± 27	-	-	-	-
21	17	62 ± 29	-	-	-	-
22	15	70 ± 29	-	-	-	-
23	15	62 ± 30	-	-	-	-
24	14	61 ± 30	-	-	-	-
25	13	68 ± 30	-	-	-	-
26	13	60 ± 30	-	-	-	-
27	12	63 ± 26	-	-	-	-
28	12	59 ± 26	-	-	-	-
